# Attenuation and soil biodegradation of fungicides by using vegetated buffer strips in vineyards during a simulated rainfall–runoff event

**DOI:** 10.1007/s11356-023-27766-9

**Published:** 2023-06-22

**Authors:** Paula Ortega, Mònica Escolà Casas, Emilio Gil, Víctor Matamoros

**Affiliations:** 1 Department of Agro-Food Engineering and Biotechnology, Universitat Politècnica de Catalunya, Esteve Terradas, 8, 08860 Castelldefels, Spain; 2 Department of Environmental Chemistry, IDAEA-CSIC, Jordi Girona, 18-26, 08034 Barcelona, Spain

**Keywords:** Copper, Fungicides, Vineyard, Runoff, Buffer zones, Buffer strip, TPs

## Abstract

Rainfall–runoff events occurring in vineyard fields can result in pesticide ground losses and the subsequent pollution of surface water bodies, derivate from the crop protection spray applications. In this study, the capacity of vegetated buffer strips (BS) to prevent surface water pollution due to the application of five fungicide products typically used in vineyards (copper, dimethomorph, oxathiapiprolin, zoxamide, acibenzolar-s-methyl, and laminarin) following a simulated run-off event has been assessed, and compared to that from a bare ground soil (BG). Two strips (5 m in length, each), one with vegetation and the other without were built up, and two different experiments were performed, a runoff event and a soil fungicide degradation kinetic evaluation. The runoff results show that fungicide mass retention in the strips ranged from 73 to 98% and that the presence of vegetation in BS increased the fungicide mass retention in the strips by almost 10% (on average) in comparison to the unvegetated strip. Moreover, soil degradation studies highlighted that the presence of vegetation reduces significantly the half-time life of almost all the studied fungicides by 55%, on average. Eight fungicide transformation products (TPs) were identified following a runoff event in the soil strips, but the abundance of these TPs was up to 78% lower in vegetated strips. These results highlight the effectiveness of using vegetated buffer zones in vineyards to protect aquatic ecosystem pollution.

**Supplementary Information:**

The online version contains supplementary material available at 10.1007/s11356-023-27766-9.

## Introduction

The use of pesticides for crop protection in vineyards is an essential part of today’s agricultural production system. As a result, the runoff of pesticides following a rainfall event has been identified as one of the most important sources of pesticide pollution into surface water bodies (Reichenberger et al., 2007, Freitas et al., 2008; Frey et al., 2009; Wohlfahrt et al., 2010). However, despite fungicides being the main pesticides used in viticulture, only limited studies have been conducted to investigate their behaviour and fate following a run-off event (Lefrancq et al., 2014). Lefrancq et al. (2017) reported the occurrence of fungicides such as dimethomorph in the runoff water of a vineyard plot with concentrations up to 13 μg L^-1^ among others, posing a risk for aquatic organisms. Moreover, in vineyards, the traditional use of fungicides against downy mildew (*Plasmopara viticola*), such as copper-based products, has resulted in a negative effect on soil organisms (Ballabio et al., 2018).

In view of this, the European Commission (EC) has launched the European Green Deal programme (EC, 2019), with the aim of changing the current agricultural production model to one that is more environmentally friendly. This programme includes several focal points for action, including the “farm-to-fork” strategy (EC, 2020). This initiative aims to turn the European agricultural system into a more sustainable sector which reduces the environmental impact and increases at the same time the food quality level. To this end, a number of targets have been set, including a 50% reduction in the overall use of chemical pesticides, and in this way contribute to reducing the environmental pollution. Furthermore, the Sustainable Use Directive of Pesticides (2009/128/EC) establishes a framework for European agriculture to achieve a more sustainable use of pesticides and reduce the risks to human health and the environment that derivate from the use of these products. In the case of copper, it has become a major environmental and toxicological problem in vineyards and EU legislation has been adopted to limit its use (Commission Implementing Regulation EU 2018/1981).

In this frame, the EU established the use of buffer zones to protect non-target organisms and safeguard water bodies from pesticide spray drift, drain flow and runoff (Directive 2009/128/EC), and also training and awareness-raising projects for farmers have also been carried out (http://www.topps-life.org/). However, the use of vegetation in those zones is not mandatory. Buffer strips are linear bands of permanent vegetation adjacent to an aquatic ecosystem intended to minimize the pollution from diffuse sources by trapping the pollutants (Barling and Moore, 1994; Mancuso et al., 2021) and have therefore been found to be an effective way of reducing the transfer of pesticides caused by surface runoff from fields to watercourses (Aguiar et al., 2015; Chen et al., 2019; Lacas et al., 2005; Otto et al., 2008). For example, at the EU level, the FOCUS working group on landscape mitigation factors recommends assuming 50%, 75%, and 90% runoff reduction for 5-m, 10-m, and 15–20-m wide buffer strips (FOCUS, 2007), respectively (Ohliger and Schulz, 2010). Nevertheless, most of the pesticide runoff studies focus on herbicides, even in vineyard fields (Jurado et al., 2012), and the results from the use of buffer strips for reducing fungicide run-off in vineyards seem to be contradictory. For instance, Bereswill et al. (2012) observed that there is no substantial difference between buffer strip width and the reduction of fungicides such as copper or dimethomorph. Furthermore, the application of organic fungicides on agricultural soils can also lead to the generation of transformation products (TPs) (Menger et al., 2021), which may have the same or greater toxicological effects on watershed ecosystems than parental fungicides (Iwafune, 2018; Meffe et al., 2021). Currently, none of the studies performed until now has addressed the soil degradation of fungicides in buffer strips nor assessed the impact of vegetation on that. Nevertheless, a previous study carried out by Ortega et al. (2021) demonstrated the capacity of using cover crops to reduce groundwater pollution by fungicides. However, there is still a lack of understanding regarding how the use of buffer strips can effectively reduce fungicide surface runoff and the subsequent fate of these compounds in the soil within the buffer strips.

This work, therefore, aims to reveal the capability of using buffer strips for the reduction of organic and inorganic fungicide pollution following a simulated rainfall–runoff event in vineyards and to assess, for the first time, the behaviour and fate of fungicides retained in the buffer strips by monitoring their TPs.

## Materials and methods

### Experimental set-up

#### Buffer strip set-up

Trials were conducted in the greenhouse facilities of the Agropolis Research Centre from the Polytechnic University of Catalonia (UPC, Viladecans, Spain) in May 2022. Two filter strips of 5.00 × 0.30 × 0.15 m (length × width × height) were built to simulate semi-field conditions buffer zones, according to Franco et al. (2016). They consisted of a 1% slope and an exit to collect samples at the end. The buffer strips were filled with 80 kg of vineyard soil (sized < 2 mm) from a commercial vineyard field located in the Tarragona region of Catalonia (Spain). The soil had a loam texture (51.2% sand, 30% silt, and 18.8% clay), 0.95% of total organic carbon content, pH of 8.8, and a C/N relation of 7.7 (further information is detailed in the supplementary material (SM) section).

Two types of ground management were tested, one in each channel: bare ground (BG) and buffer strip (BS), planted with a cover crop mix for a total soil coverage (*Trifolium subterraneum, Trifolium resupinatum, Trifolium michelianum, Biserrula pelecinus, Medicago polymorpha, Medicago truncatula, Hedysarum coronarium, Trifolium cherleri, Trifolium isthmocarpum, Dactylis glomerata, Lolium perenne, Lolium multiflorum*, and *Festuca rubra*). The total biomass was measured after the experiments removing the cover and drying it at 60° using a stove (TCF 400 Argo lab, Italy) until constant weight. The total weight of the vegetation at the BS was 406 g of aerial part and 292 g of roots.

#### Fungicide selection

With the aim to follow the same research line, the five fungicides used in Ortega et al. (2022) were based on previous trials carried out at OPTIMA project (Optimised Pest Integrated Management to precisely detect and control plant diseases in perennial crops and open-field vegetables, H2020 Grant Agreement N.773718, http://optima-h2020.eu/es/16219-2/ ) and according to Pugliese et al. (2021).

**Fig. 1:**
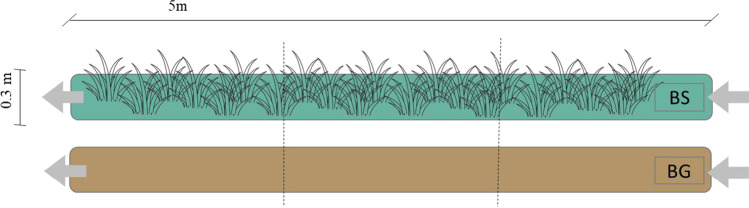
Buffer zone strips setup scheme. Arrows show the liquid flow

In fungicide application processes, there is a part that is inevitably lost to the soil. According to Gil et al. (2001), during this spraying process, for a conventional copper product (1 kg/ ha) in vineyards and under worst case conditions (an early crop stage and a conventional sprayer), the produced spray ground losses are estimated in 4.62 μg cm^-2^. Table 1 shows the estimated soil losses and the maximum dosage of the selected products according to their labels; the total soil losses were estimated for each selected product. These losses were calculated for a theoretical square hectare (Fig. 1-SM), assuming that all losses are carried over as run-off, from the field area to the constructed buffer zone (considering the same width, 0.3 m).

**Table 1:** Fungicides used in the study, their expected ground losses and log Kow : octanol–water partition coefficient

Commercial name	Active ingredient	Expected ground losses for the calculated area (mg)	Log Kow	Use
Codimur 50®	Copper oxychloride 50% (WP) P/P	416	-	Control/reference
Zorvec™ Vinabel®	Oxathiapiprolin 4 % (P/V) 40 g/L Zoxamide 30 % (P/V)	190	5.74 (Pesticide Properties DataBase /EPI Suite^TM^) 3.76 (PubChem 122087).	Novel synthetic organic fungicide with systemic action
Forum®	15.0% (p/v) dimethomorph	347	2.68 (PubChem 5889665).	Novel synthetic organic fungicide with systemic action.
Bion MX®	Acibenzolar-s-methyl 50% [WG] P/P	42	3.10 (PubChem 86412 ).	Synthetic inducer and activator of plant self-defense mechanisms
VACCIPLANT®	Laminarin 4.5% [SL] P/V	277	− 7.10 (EFSA website /EPI Suite^TM^)	Ecological organic activator of plant self-defense mechanisms

#### Effect of buffer strips on the fungicide runoff

Experimental runoff design was based on Franco et al. (2016). Two liters of fungicide-enriched water (according to the expected ground losses of Table 1, the concentration of fungicides in the enriched-water ranged from 3 to 104 mg L^-1^) were injected on the surface of each of the buffer strips by a peristaltic pump with a hydraulic loading rate (HLRs) of 1 cm h^−1^. This simulates the first runoff portion, in which the water washes the products from the field, emulating the worst case scenario in which pesticide retention in the field is not considered. Continuously, an additional 40 L of water, unenriched, was injected into each system to emulate the remaining runoff water in the field, for a total of 14 m^3^ ha^-1^, according to an average value of the field factors of area and runoff quantity (Ramos et al., 2006). Leached waster was collected at the outputs of the channels every 1 L with a total collected volume of 30 L for the BG strip and 15 L for the BS one. The collection time of each sample was recorded.

#### Effect of buffer strips on fungicide biodegradation

A soil kinetic study was performed to evaluate the degradability of the compounds in the vegetated (BS) and unvegetated (BG) buffer strips after one month of the first assay performance and taking blank samples to ensure there was no contamination. The same number of fungicides as in the previous experiment (Table 1) were applied manually on the soil surface of each buffer strip diluted in 2 L of water to distribute it uniformly. Kinetic degradation was assessed by taking 3 composite soil samples from different points and depths within each sampling section (Fig. 1). Sampling among each channel was performed at 0, 24, 72, 144, and 240 h. The buffer strips were irrigated by drip irrigation to keep the cover crops alive, but avoid leaching, with 3.2 L spread over two different times of the day.

### Analytical methodologies

For organic fungicides were followed two different procedures. Water samples were filtrated with a 0.22-μm hydrophilic PTFE filter (Frisenette, DK). Soil samples were processed as follows: 2 mL of ethyl acetate was added to 500 mg of 24 h lyophilized soil (− 20 °C and vacuum of 10^-2^ mbar), of each sample (Telstar, Madrid, Spain). The mix was ultrasonicated and centrifugated for 10 and 15 min, respectively. The liquid fraction was extracted, and the process was conducted once more. The liquid fractions were mixed and evaporated with nitrogen. Before the sample reached dryness, 500 μL of ultrapure water was added. The samples were then injected into ultra-high-performance liquid chromatograph (UHPLC) under the same conditions than Ortega et al. (2021). Specific wavelength was used for each compound (dimethomorph 235 nm, zoxamide 211 nm, acibenzolar-s-methyl 325 nm, oxathiapiprolin 258 nm, and laminarin 225 nm) and the linearity ranged from 2.5 to 2000 μg L^-1^ (LOD and LOQ of the method for the tested are specified in the Table 2-SM).

Soil sample extracts from the fungicide biodegradation experiments were analysed to find possible fungicide transformation products (TPs). To do so, one extract for each kinetic timing (0, 24, 72, 144, and 240 h) and buffer strip was analysed. The samples were run on a UPLC-QToF Impact II (Bruker Daltonics, Bremen, Germany) with a chromatographic separation matching the conditions of the instrument application TargetScreener HR. For this, we used a C18 column (Bruker Intensity Solo; *L* = 100 mm, ID = 2.1 mm, and particle size 1.8 μm; with a precolumn) operated at 40 °C. Mobile phases consisted of water/methanol (99 : 1) and methanol, both with 5 mM of ammonium formate and 0.01% of formic acid. Electrospray Ionization was performed in positive mode. Once the data was obtained, MetaboScape^®^ software (Bruker) with the inbuilt BioTransformer 3.0 (Djoumbou-Feunang et al., 2019) was used for the identification of TPs by predicting different biotic and abiotic reactions of each fungicide and comparing the resulting molecular formulas with the obtained ones. The exact mass of the predicted formula was compared to the m/z obtained masses to find matches. Among the obtained matches we only considered TPs whose exact masses were present as M+H^+^, M+Na^+^, and M+ K^+^ and had an m/z tolerance of < 3 mDa and a mSigma score < 50 with the proposed molecular formula.

On the other side, DTPA (diethylenetriaminepentaacetic acid) extraction method was used to evaluate the copper in the soil according to Lindsay et al. (1978). All copper determination was quantified with a Varian SpectrAA 110 (Mulgrave, Victoria, Australia) flame atomic absorption spectrometer, equipped with a copper hollow cathode lamp, and a deuterium lamp for background correction. The instrument was operated under the conditions recommended by the manufacturer: lamp current of 4 mA, wavelength of 324.7 nm, slit width of 0.1 nm, burner height of 14 mm, acetylene flow rate of 1.0 L min^−1^, and airflow rate of 10.0 L min^−1^ (linearity ranged from 0.5 to 5 mg L^-1^).

### Data analysis

The experimental results were statistically analysed using RStudio (RStudio Team, 2020. RStudio: Integrated Development for R. RStudio, PBC, Boston, MA URL (http://www.rstudio.com/). Kruskal-Wallis test was performed analyse statistical differences between the half-life times of the tested fungicide products in soil with and without vegetation.

## Results and discussion

### Runoff reduction and pollution mitigation in the buffer zones

**Fig. 2:**
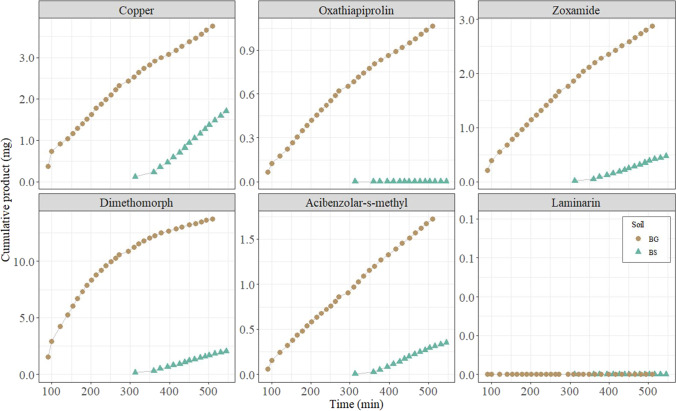
Cumulated amount of pesticides eluted by runoff for the two tested strips, bare ground (BG), and vegetated (BS)

**Fig. 3:**
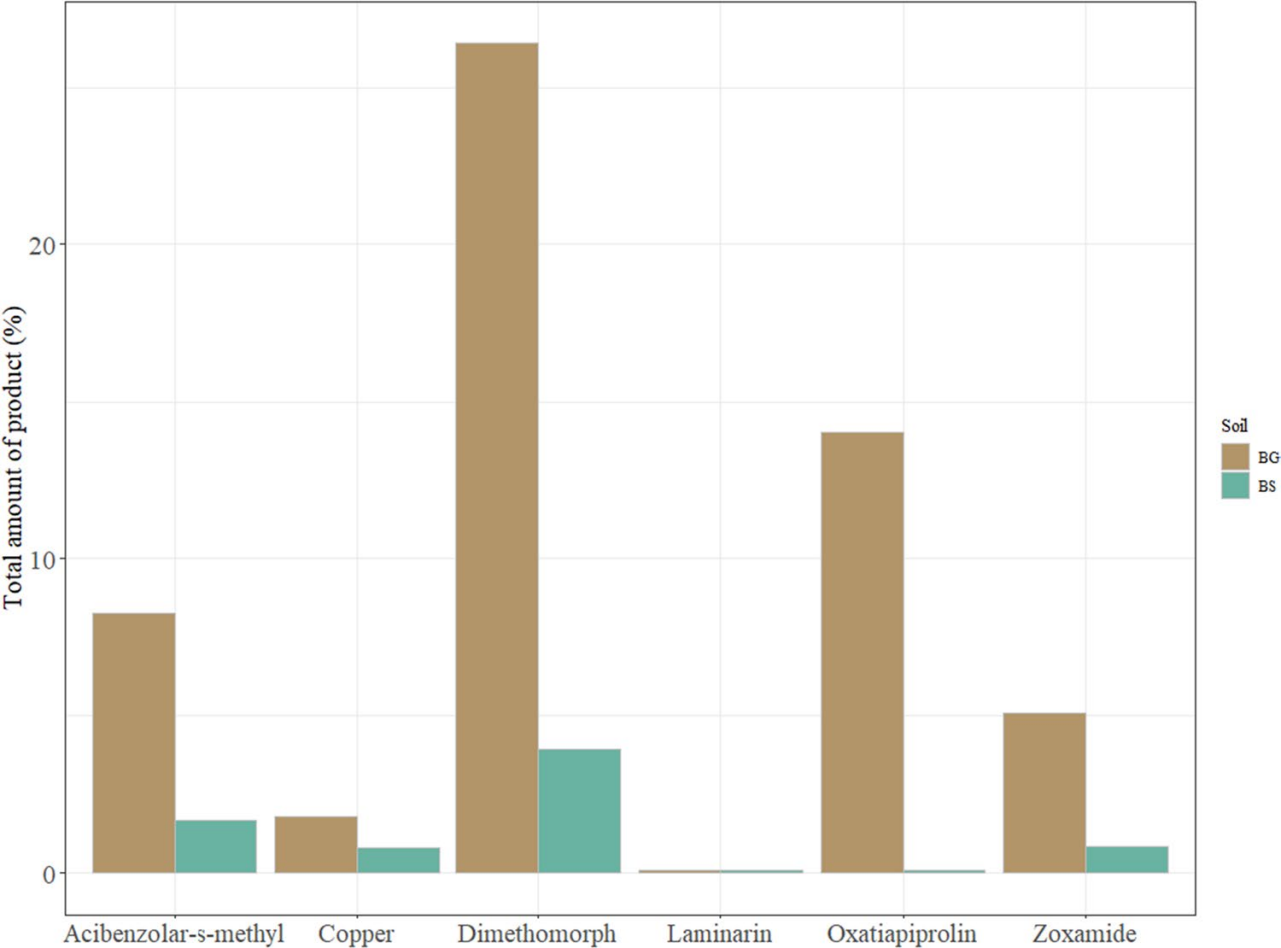
Percentage of total amount of fungicides recovered after 7 h 30 min of the runoff experiment. Bare ground (BG) and vegetated (BS)

Figure 2 shows the cumulative amount of pesticides in the final runoff water for each of the tested fungicides in the two experimental cases, bare ground (BG) and vegetated buffer strips (BS). All fungicides were detected in the final runoff water except laminarin, due to the high solubility of the compound (Table 1). The BS implies a considerable reduction of all the detected compounds in the eluted water. Furthermore, there is a clear difference between the times at which runoff water starts to elute at the end of the two strips (Fig. 2), being greater in the vegetated strip. According to Yu et al. (2016), that can be explained due to the fact that plant roots of BS significantly modify soil hydraulic conductivity and reduce the runoff from a rainfall event. Similarly, BS resulted in a lower recovery of fungicides (1–4%) than BG (1–26%), which means greater fungicide retention due to the presence of vegetation. The low recovery of fungicides (Fig. 3) in both systems can be explained by their interaction with soil (Barchańska et al., 2020), whereas plants and their rhizosphere system have been shown to be beneficial for pesticide degradation (Eevers et al., 2017). Regarding the total amount of pesticides leached at the end of the runoff study (Fig. 3), dimethomorph is the compound with the highest elution rate (with 26% in the BG and 4% in the BS) which can be explained due to its high solubility and low sorption coefficient (log Kow of 2.68). A correlation has been found with the absorbance of pesticides by roots and log Kow, which in turn correlated negatively with the translocation of these substances into the plant (Wang et al., 2017). Oxathiapiprolin compound has been leached by 14% in the BG case, but it is reduced to almost zero with the use of the cover crop (0.1 %), this is also consistent with the high reported value of the sorption coefficient and the low solubility of the compound (Table 1). Acibenzolar-s-methyl and zoxamide are also reduced, and thus their pollution potential, with the presence of the vegetation form an 8 to 2% and 5 to 0.8%, respectively. Although copper has the higher application dose and therefore the higher ground losses, the runoff simulated experiment retained 98% and only 2% of it at the outlet due to the insolubility of this compound, whereas planted buffer strip leached less than 1%. This agrees with the high sorption capacity of Cu in the soil as it has been described by (Babcsányi et al., 2016), where Cu export by runoff from the catchment in vineyard fields, accounted for 1% of the applied Cu mass. These findings are also in agreement with previous studies conducted by Ortega et al. (2021) who found that the presence of vegetation reduces fungicide groundwater pollution (covered crop soil columns had < 10% fungicide leaching, and bare soil ones had a 30% fungicide leaching). In all cases, the presence of vegetation played a very important role, reducing the runoff of organic and inorganic fungicides, and indicating a greater interaction of these compounds with the vegetation. Nevertheless, in addition to sorption, other attenuation mechanisms such as biodegradation or plant uptake cannot be ruled out.

### Fungicide soil degradation and transformation

To check out the impact of soil biodegradation in the attenuation of fungicides in the buffer strip, a first-order kinetic study was performed. Table 2 shows that the kinetic rates for the degradation of fungicides in soil ranged from 0.05 to 0.096 d^-1^ and from 0.05 to 1.54 d^-1^ in the vegetated and unvegetated strips, respectively. The variance (represented by standard deviation) of kinetic parameters values was high, probably due to the high soil heterogeneity (despite the use of soil composite samples), and especially for compounds with low mobility like copper. Dimethomorph, oxathiapiprolin, and copper had lower kinetic rates, indicating that among studied fungicides these are the most recalcitrant ones. This is in agreement with previous studies indicating that copper is a very stable compound in soil (Rehman et al., 2019), and that dimethomorph had a kinetic reduction rate of 0.034 d^-1^ in vineyard topsoil (Masbou, et al., 2022). The presence of vegetation in the BS enhanced the soil degradation kinetic rates for all the tested compounds, except for laminarin which could not be detected in any of the sampled soils. Similarly, half-lives (*t*_1/2_) for fungicide’s active ingredients were notoriously reduced by the action of vegetation. Statistically significant differences (*α* < 0.05) were observed for the oxathiapiprolin, zoxamide, and dimethomorph compounds, from a half-life of 13.3, 3.7 and 15.2 days for the BG to 3.8, 1.2, and 2.6 days for the BS, respectively. Acibenzolar-s-methyl had much lower values of half-live and the effect of the covered crop was not as noticeable as in the previous cases. As copper cannot be degraded, it can be assumed that its concentration decay in the BS was accounted to the plant uptake as a micronutrient (Yruela, 2009), thus reducing the amount of metal in the soil. To a greater or lesser extent, the presence of vegetation in BS reduced fungicides half-lives in soil. Our results are in agreement with previous studies that indicate that the presence of plants decreases the amount of pesticides in the soil due to phytoremediation processes such as phytoextraction, phytodegradation, or phytostimulation, among other occurring processes (Pascal-Lorber and Laurent, 2011; Tarla et al., 2020). For instance, Chen et al. (2018) observed that dimethomorph had similar values of 1.7 to 3.8 days with potato crops and 11.5–18.5 days in bare soil, which agrees with the results of this study. Comparing these results (Table 2) with previous ones where the impact of vegetation was assessed for the same compounds under hydroponic conditions (Ortega et al., 2022), the kinetic rates in soil are greater for all compounds, except for oxathiapiproline, which remains the same. This suggests that soil enhanced the development of bacteria in the rhizosphere as well as the biodegradation of fungicides.

**Table 2.** First-order kinetics parameters for the removal of fungicides in soil. Kruskal–Wallis (*p* < 0.05) (*n* = 3)

Product	Soil	*k* (d^-1^)	***R***^2^	***t*½ **(d)	
Copper	BG	0.05 ± 0.05	0.59	24 ± 15	
	BS	0.05 ± 0.03	0.84	15.9 ± 8.3	
Oxathiapiprolin	BG	0.08 ± 0.06	0.95	13 ± 8.9	*
	BS	0.21 ± 0.10	0.75	3.8 ± 1.5	
Zoxamide	BG	0.22 ± 0.12	0.84	3.7 ± 1.7	*
	BS	0.58 ± 0.16	0.79	1.2 ± 0.29	
Dimethomorph	BG	0.05 ± 0.01	0.66	15 ± 5.3	*
	BS	0.33 ± 0.16	0.64	2.6 ± 1.6	
Acibenzolar-s-methyl	BG	0.96 ± 0.01	0.52	0.7 ± 0.1	
	BS	1.54 ± 0.95	0.78	0.5 ± 0.3	
Laminarin	BG	-	-	-	-
	BS	-	-	-	-

*Values are statistically different at a *p* value of 0.05

### Identification of transformation products (TPs) and their behavior on BG and BS soils

**Fig. 4:**
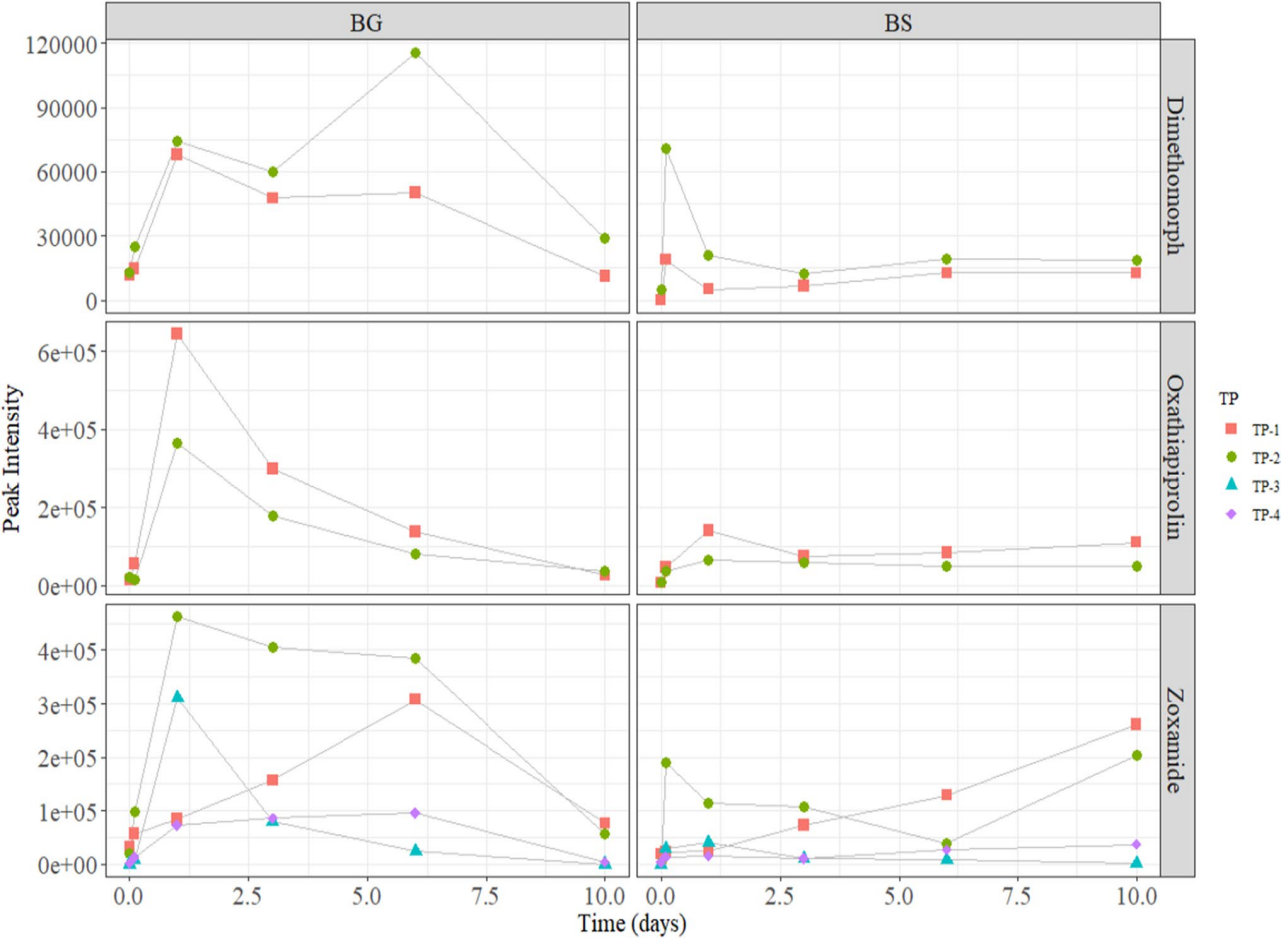
Transformation products (TPs) detected into the soil for the different tested compounds in the bare ground (BG) and buffer strip (BS)

The fungicide concentration decay over time observed in the soil was linked to the identification of several TPs, which as indicated in the introduction can have a similar or greater toxicological impact. Their molecular formulas, retention times, and matching literature structures are shown on Table 3-SM. For dimethomorph, we identified two TPs that were the result of an oxidation and a demethylation, respectively (dimethomorph TP1 and TP2, C_21_H_22_ClNO_5_, and C_20_H_20_ClNO_4_, Fig. 4a). From these, the estimated molecular formula of TP2 matched with that of two common dimethomorph soil metabolites (Z67 and Z69) (Lewis et al., 2016, David Lunn, n.d.). Oxathiapiprolin generated two other TPs with the same molecular formula. These were two different oxidation products (Oxathiapiprolin TP1 and TP2, C_24_H_22_F_5_N_5_O_3_S, Fig. 4b). Such molecular formula also matched with that of two common soil metabolites of oxathiapiprolin (IN-RDT31 and IN-RDG40) ((EFSA) et al., 2022). For zoxamide, we found four TPs. Two of the TPs underwent oxidative dechlorination (zoxamide TP1 and TP2, C_14_H_17_Cl_2_NO_3_, Fig. 4c). Their molecular formula corresponds to that of an already identified metabolite of zoxamide (RH-150721) ((EFSA) et al., 2017). The other two TPs underwent oxidative dechlorination and hydrogenation (Zoxamide TP3 and TP4, C_14_H_19_Cl_2_NO_3_, Fig. 4c). No TPs were identified for acibenzolar-s-methyl. Laminarin or its TPs could not be analyzed.

Figure 4 shows the formation of all TPs on the soil extracts from the fungicide soil biodegradation experiments. In general, BG soil resulted in higher relative amounts of the identified TPs than the BS soil, suggesting that vegetation enhanced degradation of both parent fungicides and their TPs (Escher and Fenner, 2011). In the case of dimethomorph, the concentrations of its TPs on the BG soil rose or remained stable until day 6 and then they decreased until day 10. In contrast, in the BS soil, dimethomorph TP concentrations were maintained very low over all the experiment (Fig. 4a). Almost the same behavior was observed for oxathiapiprolin. While the two TPs were formed within 1 d of exposure and then their concentrations decreased until day 10, the same TPs remained very low on the BS soil over the whole experimental period (Fig. 4b). These patterns seem to indicate that plants and their rhizosphere on BS were able to uptake or to metabolize the TPs while they were being formed and accumulated in the soil without vegetation. All TPs behaved as the TPs of dimethomorph and oxathiapiprolim, meaning that they were being formed between days 1 and 6 and then degraded until day 10, except zoxamide TP1, TP2 and TP4. These TPs, unlike the other fungicide, were being formed in BS soil over time; reaching concentrations somewhat higher than on BG at day 10 (Fig. 4c). This may indicate that for these TPs there was a plant uptake and biodegradation equilibrium which was modified over time (Meffe et al., 2021).

All the identified TPs were maintaining the core structure of the parent fungicide. Therefore, these TPs could still maintain some of their biological activity. In this sense, BS soil showed to contain lower amounts of TPs than BG soil, especially when looking at span-times up to 6 days. As a result, the use of BS is not only beneficial to degrade fungicides but also to minimize the amounts of potentially toxic TPs.

Overall, the study indicates that the presence of vegetation enhances soil biodegradation of fungicides and their TPs. Although plant uptake cannot be ruled out, it has been reported to be low (Margenat et al., 2018), suggesting that vegetation could be used for animal feeding or other purposes in a safe way.

## Conclusions

This study highlights the multiple benefits of implanting vegetated buffer zones in vineyards. These buffers not only reduce the amount of fungicides that can potentially contaminate surface water through runoff, but also facilitate the faster degradation of  retained soil fungicides and their TPs. Even though laminarin was not detected at the final run-off of the buffer zone, the amount of copper and the tested organic fungicides was reduced, at least by half, due to the action of the vegetation in the buffer strip (retention and biodegradation enhancement). The presence of vegetation had also a significant influence on the soil degradation of the tested compounds, accelerating their kinetic removal rates (from 0.36 to 0.66 d^-1^, on average). Fungicide TPs of the analysed compounds were also mitigated by the vegetated buffer strip in comparison to the bare ground one. In view of these results, we recommend the implementation of vegetation at the buffer zones in vineyards, especially when vineyards are close to sensitive aquatic ecosystems or protected water bodies.

## Supplementary Information

ESM 1: **Fig.1-SM**. Outline of the ground losses theorical calculation area. **Table 1-SM**. Maximum label dose, expected product losses and expected product washed by the runoff of selected fungicides according to Gil et al. (2001). **Table 2-SM:** LOD and LOQ of the method for the tested compounds in ng·ml. **Fig.2-SM**. Pesticides eluted by runoff for the two tested strips, bare ground (BG) and vegetated (BS). **Table 3-SM:** TPs detected into the soil, molecular weight, m/z measured, and possible matching metabolite. **Fig.3-SM**. Close-up view of the runoff assay. **Fig.4-SM**. Close-up view of the soil assay (DOCX 1274 kb)

## Data Availability

The data generated in the present study are included in the article and available from the corresponding author on request.
