# Supplementary material for: Attenuation and soil biodegradation of fungicides by using vegetated buffer strips in vineyards during a simulated rainfall–runoff event
Source: Environ Sci Pollut Res Int. 2023 Jun 22;30(35):83678–86. doi: 10.1007/s11356-023-27766-9 (PMC10359343; doi:10.1007/s11356-023-27766-9)

To be submitted to Environmental Science and Pollution Research

Attenuation and soil biodegradation of fungicides by using vegetated buffer strips in vineyards during a simulated rainfall-runoff event

Paula Ortega^1,2^, Mónica Escolà^2^, Emilio Gil^1^, Víctor Matamoros^2^

^1^ Universitat Politècnica de Catalunya, Department of Agro-Food Engineering and Biotechnology, Esteve Terradas, 8, 08860 Castelldefels, Spain

^2^ Department of Environmental Chemistry, IDAEA-CSIC, Jordi Girona, 18-26, 08034 Barcelona, Spain

**Fig.1-SM**. Outline of the ground losses theorical calculation area

**
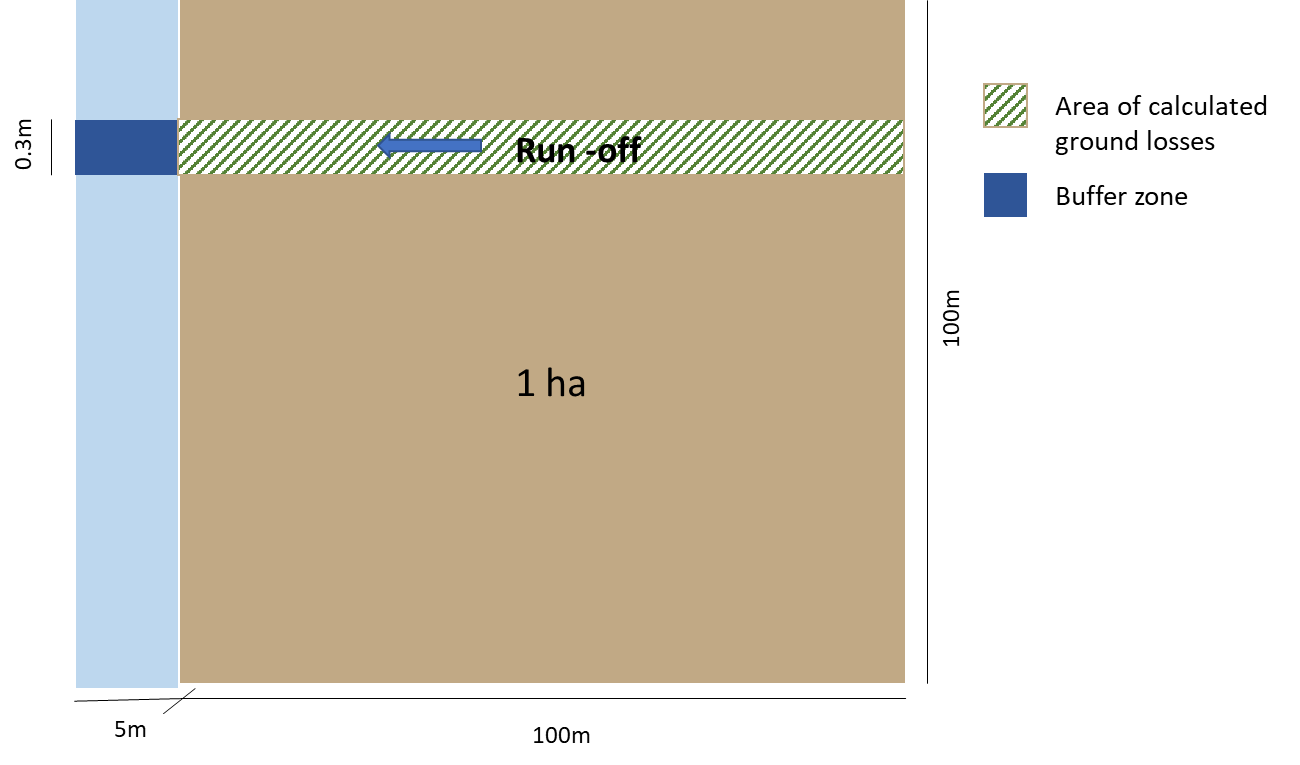
**

**Table 1-SM**. Maximum label dose, expected product losses and expected product washed by the runoff of selected fungicides according to Gil et al. (2001).

| **Commercial product name** | **Label higher dose (kg·ha^-1^)** | **Expected product losses (µg·cm^-2^)** | **Amount of ground losses for the calculated area (mg)** |
| --- | --- | --- | --- |
| CODIMUR 50  (Copper oxychloride) | 3 | 13.86 | 416 |
| ZORVEC VINABEL (Oxathiapiprolin and Zoxamide) | 1.37 | 6.33 | 190 |
| FORUM  (Dimethomorph) | 2.5 | 11.55 | 347 |
| BION MX (Acibenzolar-s-methyl) | 0.3 | 1.38 | 42 |
| VACCIPLANT (Laminarin) | 2 | 9.24 | 277 |

**Table 2****-SM:** LOD and LOQ of the method for the tested compounds in ng·ml.

| **Compound** | **LOD**  **Water (**ng·mL^-1^) **/ Soil (**ng·g^-1^) | **LOQ**  **Water (**ng·mL^-1^)**/ Soil (**ng·g^-1^) |
| --- | --- | --- |
| Oxathiapiprolin | 6.32 / 27.37 | 6.80 / 28.40 |
| Zoxamide | 2.13 / 6.63 | 1.30 / 7.44 |
| Dimethomorph | 2.13 / 13.15 | 2.64 /15.49 |
| Acibenzolar-s-metil | 4.32 / 6.51 | 5.91/ 6.95 |
| Laminarin | 1.00 | 1.37 |

**Fig.2-SM**. Pesticides eluted by runoff for the two tested strips, bare ground (BG) and vegetated (BS).


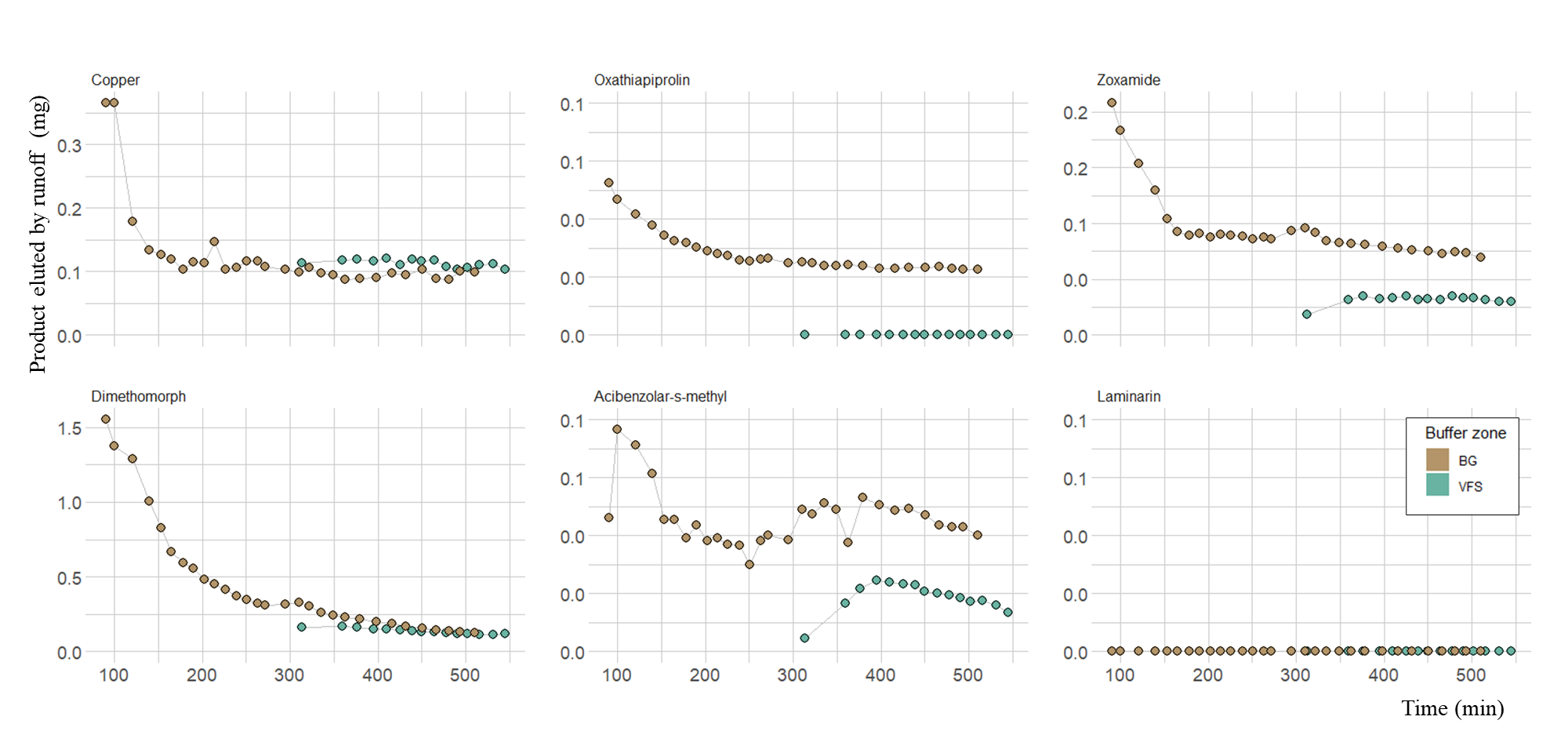


**Table 3-SM:** TPs detected into the soil, molecular weight, m/z measured, and possible matching metabolite.

| **Transformation product** | **RT**  **(min)** | **Molecular formula** | **m/z measured** | **Possible matching metabolite from literature** |
| --- | --- | --- | --- | --- |
| Dimethomorph TP1 | 8.64 | C_21_H_22_ClNO_5_ | 404.12608 | - |
| Dimethomorph TP2 | 8.74 | C_20_H_20_ClNO_4_ | 374.11557 | Dimethomorph metabolite Z67:  (4-[(E)-and(Z)-beta-(p-chlorophenyl)-3-hydroxy-4-methoxycinnamoyl]morpholine)  Dimethomoprh metabolite Z69:  (4-[(E)-and(Z)-beta-(p-chlorophenyl)-4-hydroxy-3-methoxycinnamoyl]morpholine) |
| Oxathiapiprolin TP1 | 8.64 | C_24_H_22_F_5_N_5_O_3_S | 555.13731  556.14402 | Oxathiapiprolin metabolite IN-RDT31:  (1-(4-{4-[(5RS)-5-(2,6-difluorophenyl)-4,5-dihydro-1,2-oxazol-3-yl]-1,3-thiazol-2-yl}-4-hydroxypiperidin-1-yl)-2-[5-methyl-3-(trifluoromethyl)-1H-pyrazol-1-yl]ethanone)  Oxathiapiprolin metabolite IN-RDG40:  (1-(4-{4-[(5RS)-5-(2,6-difluoro-3-hydroxyphenyl)-4,5-dihydro-1,2-oxazol-3-yl]-1,3-thiazol-2-yl}piperidin-1-yl)-2-[5-methyl-3-(trifluoromethyl)-1H-pyrazol-1-yl]ethanone) |
| Oxathiapiprolin TP2 | 8.93 | C_24_H_22_F_5_N_5_O_3_S |  |  |
| Zoxamide TP1 | 7.25 | C_14_H_17_C_l2_NO_3_ | 336.03197  318.06616 | Zoxamide metabolite (RH-150721):  (3*RS*)-3-amino-3-methyl-2-oxopentyl 3,5-dichloro-4-methylbenzoate |
| Zoxamide TP2 | 9.70 | C_14_H_17_C_l2_NO_3_ |  |  |
| Zoxamide TP3 | 8.54 | C_14_H_19_C_l2_NO_3_ | 320.08198 | - |
| Zoxamide TP3 | 9.95 | C_14_H_19_C_l2_NO_3_ | 342.06376 | - |

**Fig.3-SM**. Close-up view of the runoff assay


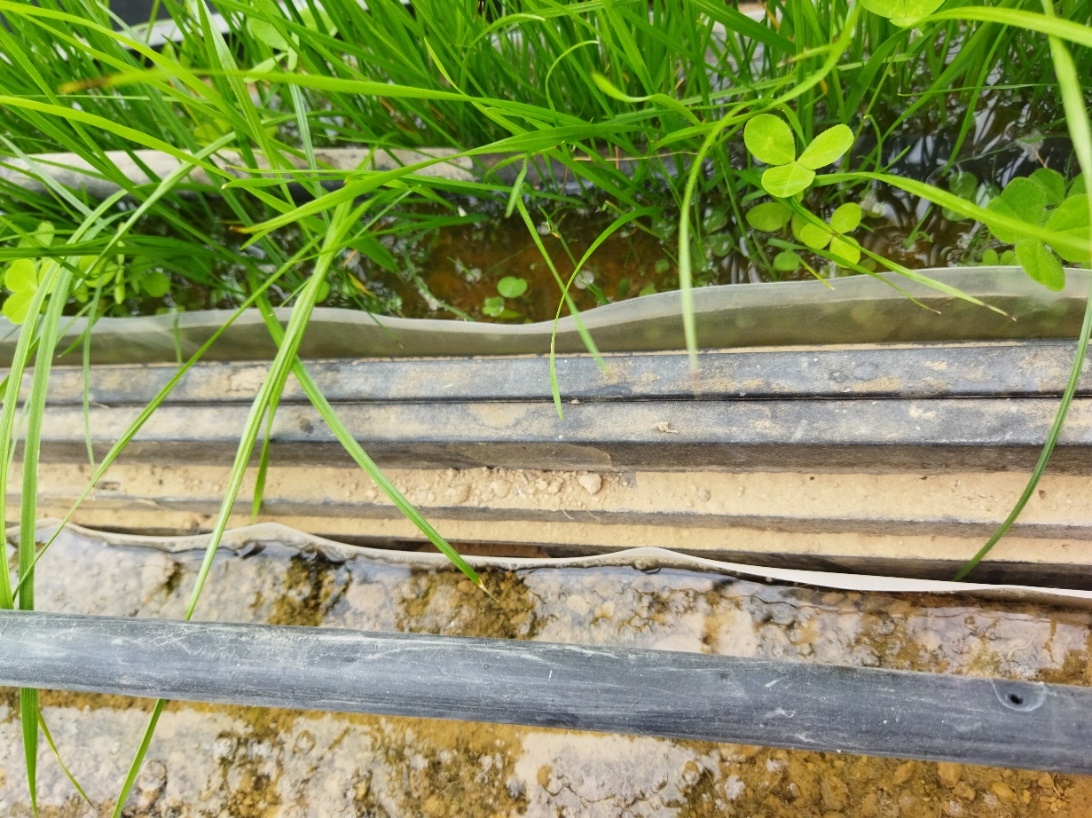


**Fig.4-SM**. Close-up view of the soil assay


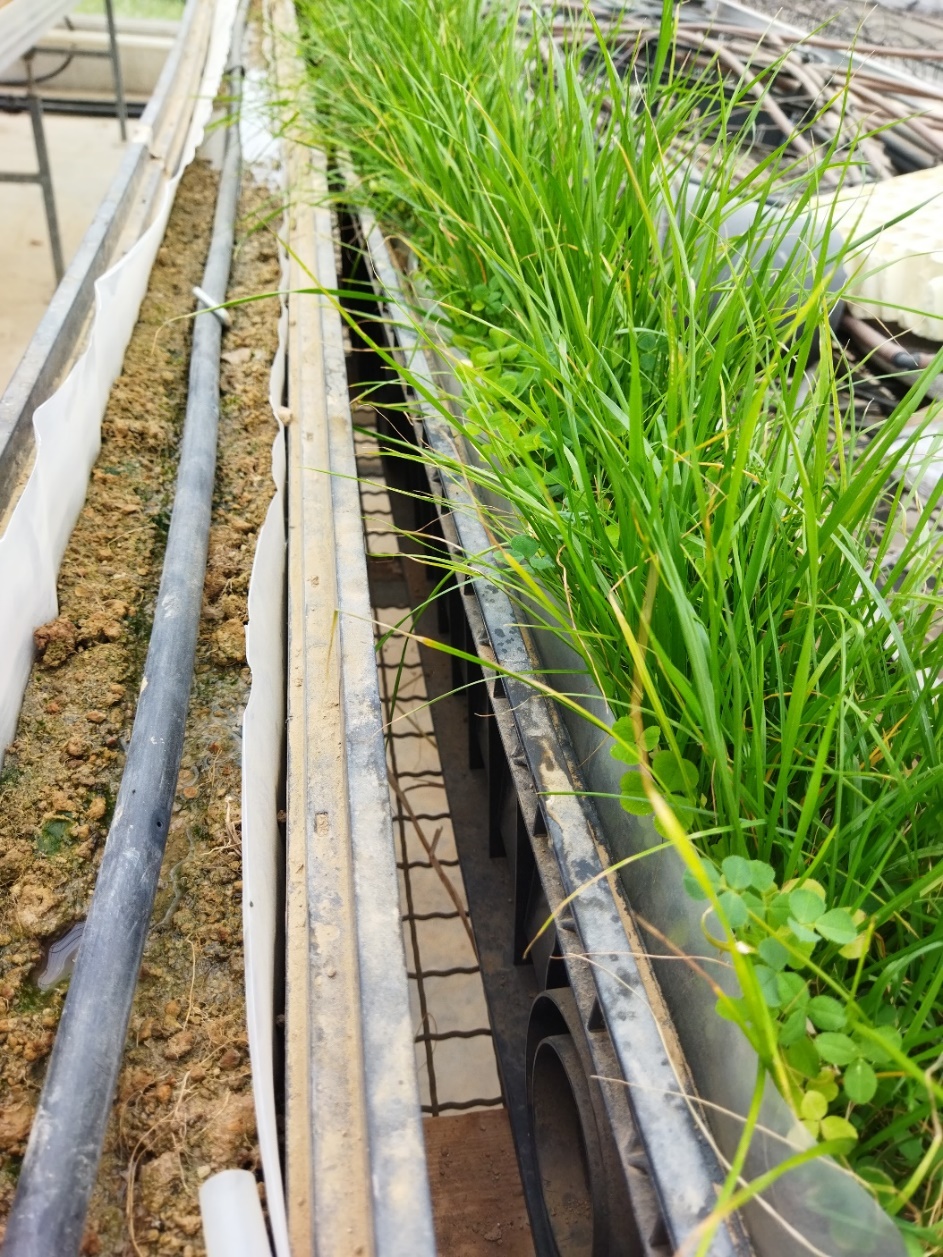

Supplement: Supplementary file 1 — Fig.1-SM. Outline of the ground losses theorical calculation area. Table 1-SM. Maximum label dose, expected product losses and expected product washed by the runoff of selected fungicides according to Gil et al. (2001). Table 2-SM: LOD and LOQ of the method for the tested compounds in ng·ml. Fig.2-SM. Pesticides eluted by runoff for the two tested strips, bare ground (BG) and vegetated (BS). Table 3-SM: TPs detected into the soil, molecular weight, m/z measured, and possible matching metabolite. Fig.3-SM. Close-up view of the runoff assay. Fig.4-SM. Close-up view of the soil assay (DOCX 1274 kb) [file 11356_2023_27766_MOESM1_ESM.docx]
